# Supplementary material for: New Gall-Forming Insect Model, Smicronyx madaranus: Critical Stages for Gall Formation, Phylogeny, and Effectiveness of Gene Functional Analysis
Source: Insects. 2024 Jan 16;15(1):63. doi: 10.3390/insects15010063 (PMC10816246; doi:10.3390/insects15010063)
Supplement: Supplementary file 1 [file insects-15-00063-s001.zip › FigS1.pdf]

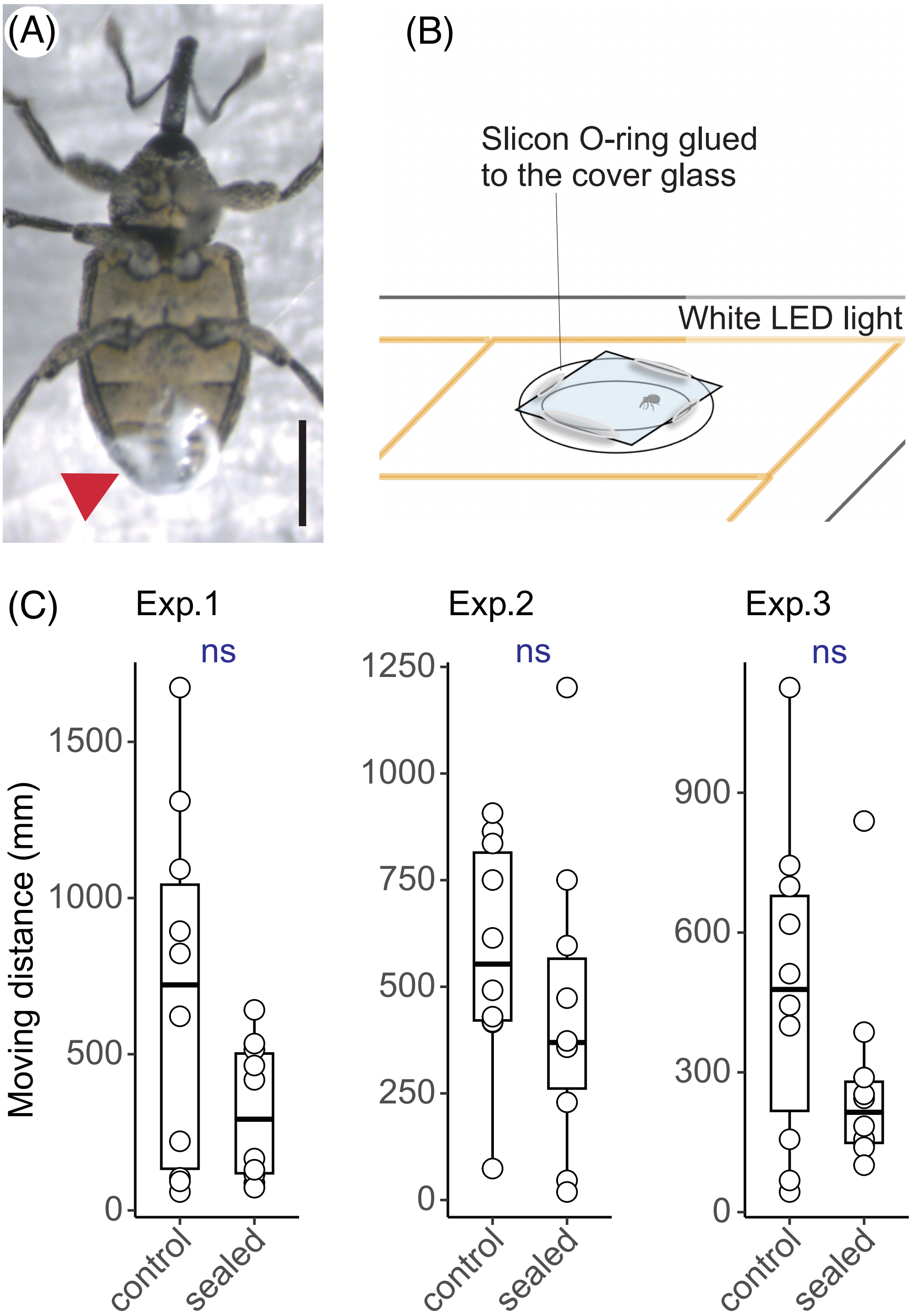

Figure S1. Effects of sealed ovipositors on behavioral activity. (A) Adult female with ovipositor sealed by white nail polish (arrowhead). Bar, 1 mm. (B) Illustration of a container for observing locomotor activity on a white LED light. (C) Behavioral activity recorded in three experiments (Exp.1–3). Box plot represents the distribution of the moving distance of control and sealed-ovipositor groups. Each open circle indicates the value of one female. ns indicates no significant difference (Wilcoxon rank sum test).
