# Supplementary material for: New Gall-Forming Insect Model, Smicronyx madaranus: Critical Stages for Gall Formation, Phylogeny, and Effectiveness of Gene Functional Analysis
Source: Insects. 2024 Jan 16;15(1):63. doi: 10.3390/insects15010063 (PMC10816246; doi:10.3390/insects15010063)
Supplement: Supplementary file 1 [file insects-15-00063-s001.zip › FigS2.pdf]

```
SmLac2A  MWSFRKCLPATVALLAVCLVPSVYTVRVGGGSRGATRKGDQDAAASWNSWDAIEPDFFRSEQQVLQTHPI  70
SmLac2B  MWSFRKCLPATVALLAVCLVPSVYTVRVGGGSRGATRKGDQDAAASWNSWDAIEPDFFRSEQQVLQTHPI  70

SmLac2A  RGNGQVSYSGSSGGGLKHKHEDFRTSPTAELRRNPSLSSPDECARACREGEPPRICYYHFTLEFYTVLGA  140
SmLac2B  RGNGQVSYSGSSGGGLKHKHEDFRTSPTAELRRNPSLSSPDECARACREGEPPRICYYHFTLEFYTVLGA  140

SmLac2A  ACQVCTPNATNAIWSNCQCILADGVERGMLAVNRMLPGPSIQVCENDKVVIDVENHMEGMEVTIHWHGIW  210
SmLac2B  ACQVCTPNATNAIWSNCQCILADGVERGMLAVNRMLPGPSIQVCENDKVVIDVENHMEGMEVTIHWHGIW  210

SmLac2A  QRGSQYYDGVPFVTQCPIQQGNAFRYQWIAGNAGTHFWHAHTGLQKMDGIYGSIVVRQPPNRDPNSNLYD  280
SmLac2B  QRGSQYYDGVPFVTQCPIQQGNAFRYQWIAGNAGTHFWHAHTGLQKMDGIYGSIVVRQPPNRDPNSNLYD  280

SmLac2A  FDLTTHVILLSDWMHENAAERFPGRLAVNTGQDPENLLINGKGQFRDPNTGFMTNTPLEIFTMTPGKRYR  350
SmLac2B  FDLTTHVILLSDWMHENAAERFPGRLAVNTGQDPENLLINGKGQFRDPNTGFMTNTPLEIFTMTPGKRYR  350

SmLac2A  FRMINSLASVCPAQLTIQGHTLTLIATDGEPVHPVNVNTIISFSGERYDFVINADQAVGAYWIQVRGLGE  420
SmLac2B  FRMINSLASVCPAQLTIQGHTLTLIATDGEPVHPVNVNTIISFSGERYDFVINADQAVGAYWIQVRGLGE  420

SmLac2A  CGIRRVQQLGILRYYKGPYTPFTQAPTYDFGIAQGVVLNPLDARCNETRADAVCISQLKNAREVDRALLI  490
SmLac2B  CGIRRVQQLGILRYYKGPYTPFTQAPTYDFGIAQGVVS------------------------------IV  460

SmLac2A  EKPNVKIFLPFRFHVYTPQDLFAPNTYNRHLVAPNGDHVISLVDEISYMAPPAPLISQYDDTDPEQFCNG  560
SmLac2B  VKKVMVFFSLSECDCLESIGLVEENTHN------------------------------------------  488

SmLac2A  DNKPANCGPNCMCTHKIDIPLNAIVEVVLVDEVQQPNLSHPFHLHGYAFNVVGIGRSPDQNVKKINLKHA  630
SmLac2B  ----------------------------------------------------------------------  488

SmLac2A  LDLDRRGLLSRHFDLPPGKDTIAVPNNGYVIFRFRADNPGFWLFHCHFLFHIVIGMNLVLQVGTHADLPP  700
SmLac2B  ----------------------------------------------------------------------  488

SmLac2A  VPLNFPTCGDHTPRINLDPTKI  722
SmLac2B  ----------------------  488
```

**Figure S2**. Alignment of the putative amino acid sequences of *SmMCO2A* and *SmMCO2B*. Dashes in the sequences represent gaps introduced to optimize alignment. The number to the right indicates the position of the last amino acid in the row. Identical residues in all sequences are black-bordered; the cysteine-rich consensus region in the insect laccase protein is in red box; presumptive signal peptides predicted by SignalP 5.0 [63] are underlined in red.
