# Supplementary material for: New Gall-Forming Insect Model, Smicronyx madaranus: Critical Stages for Gall Formation, Phylogeny, and Effectiveness of Gene Functional Analysis
Source: Insects. 2024 Jan 16;15(1):63. doi: 10.3390/insects15010063 (PMC10816246; doi:10.3390/insects15010063)
Supplement: Supplementary file 1 [file insects-15-00063-s001.zip › FigS3.pdf]

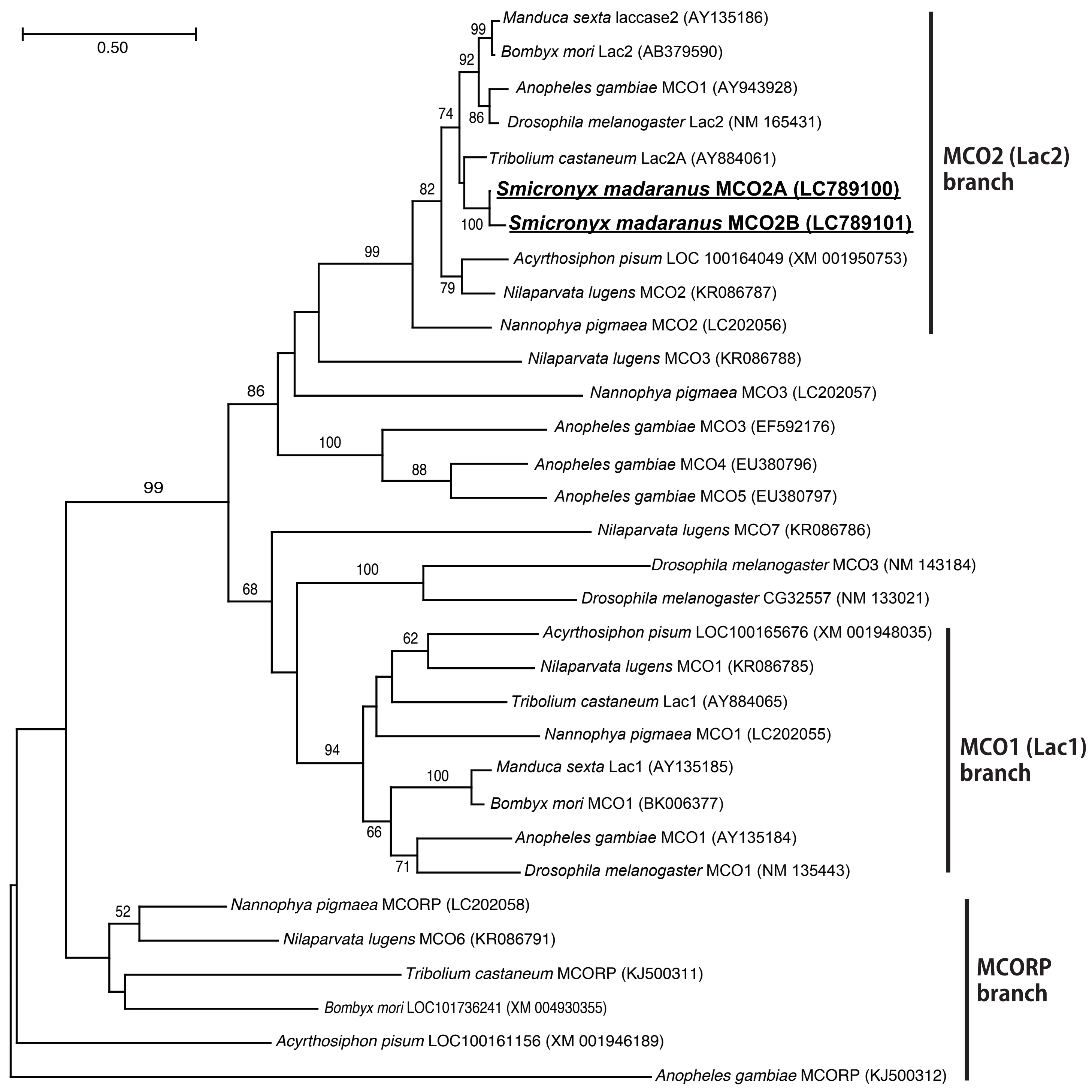

Figure S3. Molecular phylogenetic analysis of *SmMCO2* and related genes based on deduced amino acid sequences. The tree was inferred from 225 aligned amino acid sites using the maximum likelihood method. Bootstrap values based on 1,000 replications are shown as percentages on the nodes (only values ≥ 50% are shown). Sequences obtained in this study are underlined and in bold type. Sequence accession numbers are shown in brackets.
