# Supplementary material for: New Gall-Forming Insect Model, Smicronyx madaranus: Critical Stages for Gall Formation, Phylogeny, and Effectiveness of Gene Functional Analysis
Source: Insects. 2024 Jan 16;15(1):63. doi: 10.3390/insects15010063 (PMC10816246; doi:10.3390/insects15010063)
Supplement: Supplementary file 1 [file insects-15-00063-s001.zip › FigS4.pdf]

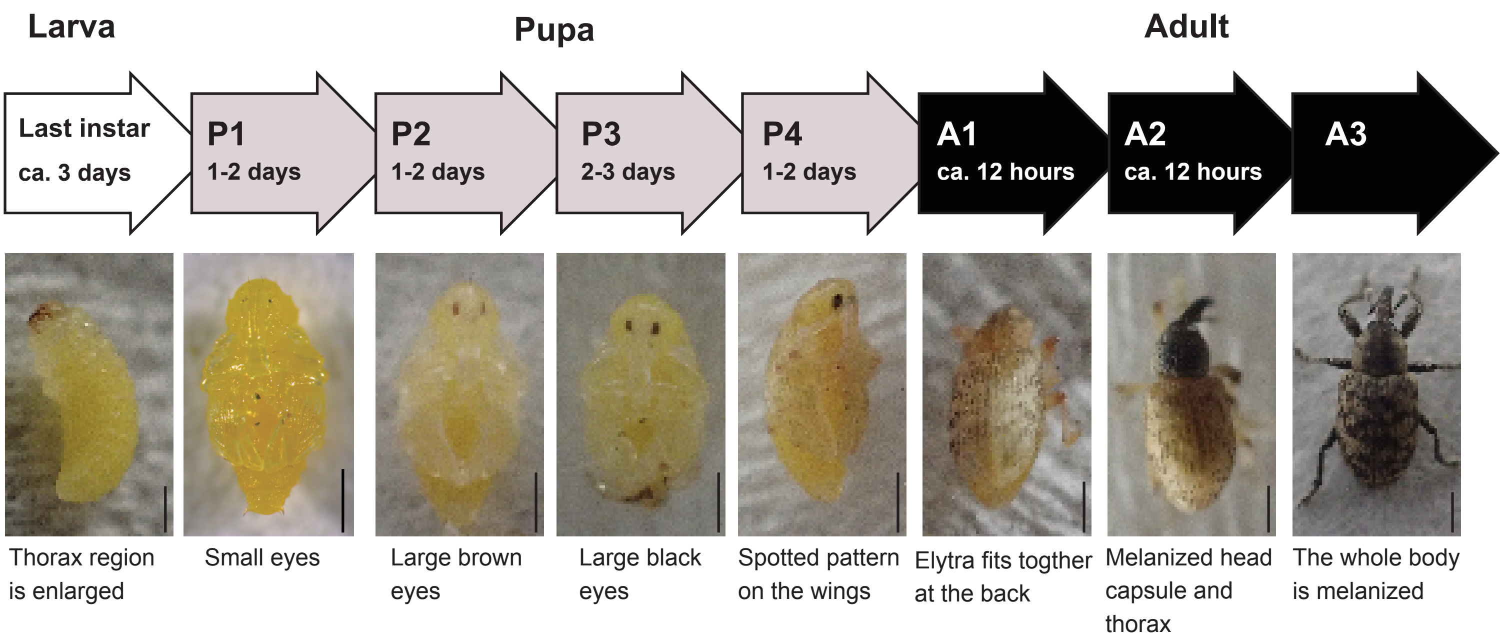

Figure S4. Growth stage and body color change in *S. madaranus*. Numbers of days on the arrow indicate the approximate durations under laboratory conditions. Bars, 1 mm.
