# Supplementary material for: New Gall-Forming Insect Model, Smicronyx madaranus: Critical Stages for Gall Formation, Phylogeny, and Effectiveness of Gene Functional Analysis
Source: Insects. 2024 Jan 16;15(1):63. doi: 10.3390/insects15010063 (PMC10816246; doi:10.3390/insects15010063)
Supplement: Supplementary file 1 [file insects-15-00063-s001.zip › Supplemental Movie Legend.pdf]

Supplemental Movie Legend

Movie S1. Observation of the gall formation using time-lapse photography. *Nicotiana benthamiana* with the first day galls were placed in a rearing case and photographed using Camera Module v.2.1 connected to Raspberry Pi 3 Model B (Raspberry Pi Foundation) [Growth condition: temperature 28 ˚C in the long day regimen (14L10D)]. Photographs were taken every 10 minutes using an original source code written in shell script. The acquired images were used to create a movie consisting of 60 frames per second using Spyder ver. 4.15 (https://www.spyder-ide.org) and FFmpeg (https://www.ffmpeg.org).

Movie S2. Digital video tracking of *S. madaranus* locomotor activity. Weevil behavior was recorded using a Raspberry Pi camera for 5 min. The video is 20 times faster than the actual speed. The behavioral traces were overlaid with an original script written in the R language based on the tracking data. Ovipositor-sealed and control groups are shown in red and blue, respectively.
