# Supplementary material for: New Gall-Forming Insect Model, Smicronyx madaranus: Critical Stages for Gall Formation, Phylogeny, and Effectiveness of Gene Functional Analysis
Source: Insects. 2024 Jan 16;15(1):63. doi: 10.3390/insects15010063 (PMC10816246; doi:10.3390/insects15010063)
Supplement: Supplementary file 1 [file insects-15-00063-s001.zip › TableS1.pdf]

Table S1 Weevil samples used in this study

Species	Locality (Collector[a])	Sample code[b]	Insect life stage (Host plant[c])	Sampling date	Accession number
Smicronyx dentirostris					
	Notsuharu, Oita, Oita (YT)	Sd_Oita 1	Adult (Cj)	2019-08-11	LC778267
		Sd_Oita 2	Adult (Cj)	2019-08-11	LC778268
Smicronyx japonicus					
	Shimizu, Shizuoka, Shizuoka (HH)	Sj_Shizuoka 1	Adult (Cca)	2019-08-03	LC778264
		Sj_Shizuoka 2	Adult (Cch)	2019-08-03	LC778265
	Nakayama, Tahara, Aichi (TW)	Sj_Tahara	Adult (Cca)	2019-09-02	LC778266
Smicronyx madaranus					
	Shinya, Akita, Akita (TW)	Sm_Akita	Larva (Cca)	2018-06-23	LC778256
	Iwase-Koshi, Toyama, Toyama (TT, RM)	Sm_Toyama	Larva (Cca)	2018-06-27	LC778254
	Minami, Fuchu, Tokyo (TT)	Sm_Fuchu	Larva (Cca)	2018-07-07	LC778255
	Nachikatsuura, Higashimuro, Wakayama (TW)	Sm_Higashimuro	Larva (Cca)	2018-06-24	LC778257
	Fukube, Tottori, Tottori (TW)	Sm_Tottori	Larva (Cca)	2018-07-15	LC778253
Smicronyx rubricatus					
	Shiramine, Hakusan, Ishikawa (HK)	Sr_Hakusan 1	Adult (Cj)	2019-08-25	LC778258
		Sr_Hakusan 2	Adult (Cj)	2019-08-25	LC778263
	Shioya, Kaga, Ishikawa (HK)	Sr_Kaga 1	Adult (Cj)	2019-07-17	LC778260
		Sr_Kaga 2	Adult (Cj)	2019-07-17	LC778261
	Hirase, Shirakawa, Gifu (HK)	Sr_Shirakawa	Adult (Cj)	2019-09-25	LC778262
	Notsuharu, Oita, Oita (YT)	Sr_Oita	Adult (Cj)	2019-08-11	LC778259

[a] TW, Tatsuya Wakasugi; TT, Tsutomu Tsuchida; RM, Ryo Murakami; HK, Hideo Kawase; YT, Yuji Tsutsumiuchi; HH, Hiroshi Hayakawa. [b] The code corresponds to the sample label in molecular phylogenetic analysis shown in Fig. 4. [c] Cca, *Cuscuta campestris*; Cj, *Cuscuta japonica*; Cch, *Cuscuta chinensis*.
