# Supplementary material for: New Gall-Forming Insect Model, Smicronyx madaranus: Critical Stages for Gall Formation, Phylogeny, and Effectiveness of Gene Functional Analysis
Source: Insects. 2024 Jan 16;15(1):63. doi: 10.3390/insects15010063 (PMC10816246; doi:10.3390/insects15010063)
Supplement: Supplementary file 1 [file insects-15-00063-s001.zip › TableS2.pdf]

Table S2 Primers used in this study

Target gene	Primer name	Direction	Sequence(5'->3') [a]	Product size (bp)	PCR Tm (℃)	References
**Primers for phylogenetic analysis**						
Cytochrome c oxidase subunit I	LCO1490	Forward	GGTCAACAAATCATAAAGATATTGG	710	50	62
	HCO2198	Reverse	TAAACTTCAGGGTGACCAAAAAATCA			62
**Primers for quantitative PCR**						
SmMCO2A and *SmMCO2B*	SmMCO2_q803F	Forward	ACCATTCATTGGCACGGTAT	204	62	This study
	SmMCO2_q1006R	Reverse	GGGCGGTTGTCTAACTACGA			This study
Elongation factor 1 alpha	WevEF1a_640F	Forward	AACATGTTGGAACCCTCCAC	175	62	This study
	WevEF1a_814R	Reverse	CACCAATTTTGTAGACATCCTG			This study
**Primers for dsRNA**						
SmMCO2A and *SmMCO2B*	T7_SmMCO2_276F	Forward	GATCACTAATACGACTCACTATAGGG TTTTCGAACGTCACCCACTG	593	62	This study
	T7_SmMCO2_810R	Reverse	GATCACTAATACGACTCACTATAGGG GGGCGGTTGTCTAACTACGA			This study
EGFP	T7_EGFP_284F	Forward	GATCACTAATACGACTCACTATAGGG AGGAGCGCACCATCTTCTTC	449	62	This study
	T7_EGFP_674R	Reverse	GATCACTAATACGACTCACTATAGGG ACGAACTCCAGCAGGACCAT			This study

[a] The T7 promoter sequences are underlined.
